# Characterization of the molecular chaperone ClpB from the pathogenic spirochaete *Leptospira interrogans*

**DOI:** 10.1371/journal.pone.0181118

**Published:** 2017-07-10

**Authors:** Joanna Krajewska, Anna Modrak-Wójcik, Zbigniew J. Arent, Daniel Więckowski, Michal Zolkiewski, Agnieszka Bzowska, Sabina Kędzierska-Mieszkowska

**Affiliations:** 1 Department of General and Medical Biochemistry, University of Gdańsk, Faculty of Biology, Gdańsk, Poland; 2 Division of Biophysics, Institute of Experimental Physics, Faculty of Physics, University of Warsaw, Warsaw, Poland; 3 University Centre of Veterinary Medicine UJ-UR, University of Agriculture in Krakow, Krakow, Poland; 4 Department of Biochemistry and Molecular Biophysics, Kansas State University, Manhattan, Kansas, United States of America; University of Pittsburgh, UNITED STATES

## Abstract

*Leptospira interrogans* is a spirochaete responsible for leptospirosis in mammals. The molecular mechanisms of the *Leptospira* virulence remain mostly unknown. Recently, it has been demonstrated that an AAA^+^ chaperone ClpB (a member of the Hsp100 family) from *L*. *interrogans* (ClpB_Li_) is not only essential for survival of *Leptospira* under the thermal and oxidative stresses, but also during infection of a host. The aim of this study was to provide further insight into the role of ClpB in the pathogenic spirochaetes and explore its biochemical properties. We found that a non-hydrolysable ATP analogue, ATPγS, but not AMP-PNP induces the formation of ClpB_Li_ hexamers and stabilizes the associated form of the chaperone. ADP also induces structural changes in ClpB_Li_ and promotes its self-assembly, but does not produce full association into the hexamers. We also demonstrated that ClpB_Li_ exhibits a weak ATPase activity that is stimulated by κ-casein and poly-lysine, and may mediate protein disaggregation independently from the DnaK chaperone system. Unexpectedly, the presence of *E*. *coli* DnaK/DnaJ/GrpE did not significantly affect the disaggregation activity of ClpB_Li_ and ClpB_Li_ did not substitute for the ClpB_Ec_ function in the *clpB*-null *E*. *coli* strain. This result underscores the species-specificity of the ClpB cooperation with the co-chaperones and is most likely due to a loss of interactions between the ClpB_Li_ middle domain and the *E*. *coli* DnaK. We also found that ClpB_Li_ interacts more efficiently with the aggregated G6PDH in the presence of ATPγS rather than ATP. Our results indicate that ClpB’s importance during infection might be due to its role as a molecular chaperone involved in reactivation of protein aggregates.

## Introduction

Bacterial ClpB is a molecular chaperone belonging to the Hsp100 subfamily of AAA^+^ ATPases (ATPases associated with a variety of cellular activities) that cooperates with the DnaK chaperone system in solubilization and reactivation of aggregated proteins [1–4]. There are a number of observations indicating that the cooperation of ClpB and DnaK in protein disaggregation is species-specific [5–8].

**Fig 1:**
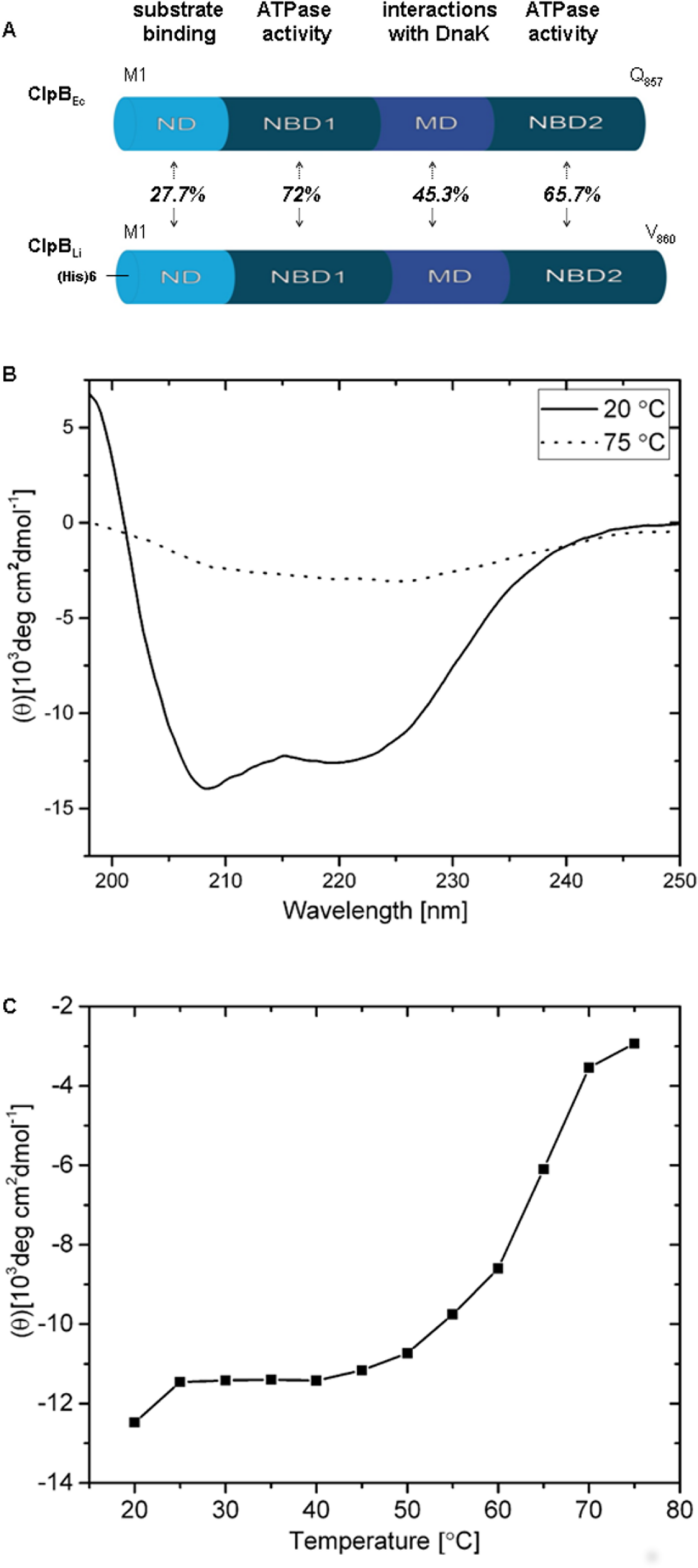
Structural characteristics of ClpB_Li_ used in this study. (A) Comparison of the domain organization of ClpB from *L*. *interrogans* and *E*. *coli*. Bacterial ClpB proteins are composed of the following domains: N-terminal domain (ND), nucleotide binding domain 1 (NBD1), middle coiled-coil domain (MD), and nucleotide binding domain 2 (NBD2). The functions of the domains are indicated at the top. The amino acid residue numbers are shown for each chaperone and the amino acid sequence identity between ClpB_Ec_ and ClpB_Li_ is indicated for each domain. (B) CD spectra of ClpB_Li_ at 20°C (folded form) and 75°C (unfolded form) are shown. The CD signal was expressed as mean molar residue ellipticity (θ). (C) Temperature-induced changes in the CD signal at 222 nm for ClpB_Li_.

Like other Hsp100 chaperones, ClpB forms barrel-shaped hexamers in the presence of nucleotides [9]. Each ClpB protomer is composed of an N-terminal domain (ND), two AAA^+^ ATP-binding modules (NBD1, NBD2), and a coiled-coil middle domain (MD) inserted at the end of NBD1 (Fig 1A). ND of ClpB is important for binding and recognition of protein substrates [10], whereas MD determines functional interactions with the DnaK chaperone system (including the apparent species-specificity of ClpB/DnaK) required for an efficient protein disaggregation *in vivo* and *in vitro* [6,7]. The mechanism of the ClpB-mediated protein disaggregation couples the ATP hydrolysis with the translocation of substrate polypeptides through the central channel of the hexameric ring [11].

ClpB plays a crucial role in survival of bacteria under stressful conditions [12,13] and is also involved in supporting virulence of some bacterial pathogens [14–17], including a pathogenic spirochaete *Leptospira interrogans* [18] responsible for leptospirosis in mammals. Leptospirosis is considered the most widespread bacterial zoonosis of global importance. More than 1 million human cases of severe leptospirosis occur worldwide each year, with up to 20% mortality rate [19]. The sources of pathogenic leptospires are mainly infected and sick animals (or asymptomatic carriers), which excrete the bacteria with urine into the environment where they can survive even for several months. Thus, water and soil contaminated with infected urine may facilitate the spread of pathogenic *Leptospira*. In moderate-climate countries, the environment is a strong risk factor for *Leptospira* infections. In many regions, including the EU, there are significant economic losses due to reproductive disorders in cattle, sheep, pigs and horses linked to leptospirosis. The disease in these species often has a latent, chronic nature. Reproductive disorders and ocular inflammation in horses are the only symptoms of the disease, which hampers diagnosis and proper treatment and generates economic losses. Many serological and microbiological studies indicate a high rate of infections in domestic animals [20–23]. Despite a severity of leptospirosis and its global importance, the molecular mechanisms of the disease pathogenesis are not well understood. Thus, identification of the *Leptospira* virulence factors and characterization of their activity is particularly important for understanding the mechanisms of the disease.

A molecular chaperone ClpB is among the few known leptospiral virulence factors [18]. However, its role in pathogenic leptospires and biochemical activity have not been investigated so far. In this study, we explored for the first time structural and biochemical properties of ClpB from *L*. *interrogans* (ClpB_Li_). As reported earlier [24], ClpB_Li_ shows a multi-domain organization similar to that of the well-characterized ClpB from *Escherichia coli* (ClpB_Ec_) (Fig 1A) and the total sequence identity between ClpB_Li_ and ClpB_Ec_ is 52%. In this study, we found that the recombinant ClpB_Li_ can assemble into hexamers in a nucleotide-dependent manner, like other well-characterized bacterial ClpBs, and shows the aggregate-reactivation activity that may support the survival of *L*. *interrogans* under the host-induced stresses. Interestingly, ClpB_Li_ may mediate disaggregation of some aggregated proteins without the assistance of the DnaK system. Furthermore, the *E*. *coli* DnaK chaperone system does not potentiate the ClpB_Li_ activity and ClpB_Li_ does not rescue the survival of *E*. *coli ΔclpB* mutant under heat shock. The apparent lack of cooperation between ClpB_Li_ and the *E*. *coli* DnaK chaperone system during aggregate reactivation *in vivo* and *in vitro* demonstrates species-specificity among the chaperones, which could have evolved to address different types of stress affecting survival of different microorganisms.

## Materials and methods

### Proteins

ClpB_Li_ was successfully overproduced as an N-terminal hexahistidine fusion protein in *E*. *coli* BL21(DE3) strain (Novagen) and then purified by immobilized metal affinity chromatography (IMAC) using Co-NTA agarose (Qiagen) and gel filtration chromatography (Superdex 200, Sigma-Aldrich) as previously reported [24]. After purification, the protein was extensively dialyzed against appropriate buffers (as described below in Materials and methods). The N-terminal histidine tag was removed by proteolytic digestion using the Thrombin Cleavage Capture Kit (Novagen) according to the manufacturer’s protocol.

*E*. *coli* chaperones (ClpB_Ec_, DnaK_Ec_, DnaJ_Ec_) were produced as previously described [25–27]. GrpE from *E*. *coli* (GrpE_Ec_) was obtained from K. Liberek (Intercollegiate Faculty of Biotechnology of UG and MUG, Gdańsk, Poland). A Zn^2+^-dependent *E*. *coli* fructose-1,6-bisphosphate aldolase (Fda) was overproduced in XL1-Blue [pKEN8 (*fda*^+^, *amp*^R^)] cells purchased from the American Type Culture Collection (ATCC 77472) and purified as described earlier [28]. Glucose-6-phosphate dehydrogenase (G6PDH) from *Leuconostoc mesenteroides*, κ-casein and poly-lysine were obtained from Sigma. Protein concentrations were estimated by the Bradford method [29] with bovine serum albumin (BSA) as a standard or from absorption at 280 nm using the extinction coefficient of ClpB_Li_ ɛ^0.1%^ = 0.445 (mg/ml)^-1^cm^-1^ calculated from the amino acid composition by ProtParam [30].

### Circular dichroism (CD) spectroscopy

The far-UV CD spectra (200–250 nm) of ClpB_Li_ at a concentration of 0.15 mg/ml were recorded in a 50 mM Tris-HCl pH 7.5, 1 mM EDTA, 1 mM DTT, 20 mM MgCl_2_, 200 mM KCl, 10% glycerol buffer, in 1-mm path-length cells, using a Jasco J-815 spectropolarimeter (Japan) equipped with Jasco Peltier element for temperature control. The mean residue ellipticity was calculated according to [31]. To assess the thermal stability of ClpB_Li_, far-UV CD signals at 222 nm were recorded between 20 and 75°C with a scan rate of 0.5°C/min. A transition mid-point temperature (T_m_) was calculated by fitting the sigmoidal Boltzman curve to the ellipticity data using the program OriginPro 9.1 (OriginLab Corp., USA, www.originlab.com).

### Sedimentation velocity analytical ultracentrifugation

Analytical ultracentrifugation was performed at 20°C with Beckman Optima XL-I analytical ultracentrifuge equipped with a four- or eight-position An-Ti rotor and UV absorption detection at 290 nm in double-sector 1.2 cm cells with charcoal-filled epon centerpieces and sapphire windows. ClpB_Li_ was dialyzed twice against 50 mM Tris-HCl pH 7.5 buffer containing 0.2 M (or 30 mM) KCl, 20 mM MgCl_2_, 1 mM EDTA, 2 mM β-mercaptoethanol, 5% glycerol, and 400 μl of the dialysate was loaded into reference sectors of the cells. Samples (390 μl) contained ClpB_Li_ (at concentrations of 1.2 or 3 mg/ml) alone or with 2 mM nucleotide: ATPγS (adenosine-5’-(γ-thio)-triphosphate); Sigma), AMP-PNP (adenosine 5′-(β,γ-imido)-triphosphate; Sigma) or ADP (Sigma). ATP analogues at the same concentrations were also added to the reference sectors. Sedimentation velocity experiments were performed at 50,000 rpm and radial absorption scans of protein-concentration profiles were measured at 4.5- or 5-min intervals. The data were analyzed using the SEDFIT program with continuous sedimentation coefficient distribution *c*(*s*) model based on Lamm equation [32]. Integration of the *c*(*s*) peaks provided the signal-weighted average sedimentation coefficients (*s*) and the corresponding standard sedimentation coefficients *s*_20,w_ (referring to water solvent at 20^°^C). Partial specific volume of ClpB_Li_ (from the amino acid composition) as well as density and viscosity of the buffer were calculated using Sednterp program [33].

### Proteolytic sensitivity assay

ClpB_Li_ or ClpB_Ec_ (1 μM) was preincubated in 50 mM Tris-HCl pH 7.5 buffer containing 200 mM KCl, 20 mM MgCl_2_, 1 mM EDTA, 1 mM DTT and 10% glycerol for 10 min on ice without or with 5 mM nucleotides: ATP, ATPγS, ADP, AMP-PNP. Trypsin (Sigma) prepared in 1 mM HCl (at a concentration of 1 mg/ml) was then added to the reaction mixtures to a final concentration of 0.2 ng/μl, and the samples were incubated at 37°C for the indicated periods (from 0 to 60 min). The reactions were quenched by the addition of Laemmli SDS-PAGE buffer and samples were analyzed by 0.1%SDS-12.5%PAGE. The gels were stained with Coomassie blue dye.

### ClpB ATPase assay

ClpB_Li_ and ClpB_Ec_ were incubated in assay buffer (100 mM Tris-HCl, pH 8.0, 1 mM DTT, 1 mM EDTA, 10 mM MgCl_2_ and 5 mM ATP) at 37°C for 30 min without or with 0.1 mg/ml κ-casein or 0.04 mg/ml poly-lysine, or 2.1 μM aggregated G6PDH. The concentration of ClpB was 0.05 mg/ml for determination of the basal activity and in the presence of κ-casein, and G6PDH or 0.005 mg/ml in the presence of poly-lysine. Inorganic phosphate concentration was determined using the malachite green dye-based colorimetric assay [34] and detection at A_640_.

All absorbance measurements in this study were performed using a model U-1900 Hitachi UV-VIS spectrophotometer.

### Aggregate reactivation assays

The purified Fda (2 μM, in buffer A: 100 mM Tris-HCl pH 7.5, 10 mM MgCl_2_ and 0.3 mM ZnCl_2_) was incubated at 55°C for 10 min. Subsequently, ATP (5 mM) and chaperones: ClpB_Li_ or ClpB_Ec_ (0.65 μM), DnaK_Ec_ (1 μM), DnaJ_Ec_ (0.2 μM) and GrpE_Ec_ (0.1 μM) were added. The total volume of a reaction mixture was 50 μl. The Fda activity was determined as described by Sigma Quality Test Procedure [35], and the decrease in A_340_ was measured after 60- and 120-minute incubation at 25°C using a spectrophotometer. The aggregated Fda in buffer A with 5 mM ATP, but without the chaperones was used as control.

Aggregates of G6PDH were prepared as described earlier [36]. The stock protein (420 μM) was diluted 2-fold with the unfolding buffer (10 mM urea, 16% glycerol, 40 mM DTT) and incubated at 47°C for 5 min. Subsequently, the mixture was diluted 10-fold by the addition of refolding buffer B (50 mM Tris-HCl pH 7.5, 20 mM Mg(OAc)_2_, 30 mM KCl, 1 mM EDTA, and 1 mM β-mercaptoethanol), incubated at 47°C for 15 min and then on ice for 2 min (stabilization of aggregates). Aggregated G6PDH (21 μM) was further diluted 10-fold with refolding buffer B. Subsequently, chaperones: ClpB_Li_ or ClpB_Ec_ (0.65 μM), DnaK_Ec_ (1 μM), DnaJ_Ec_ (0.2 μM), GrpE_Ec_ (0.1 μM) and 5 mM ATP were added. The G6PDH activity was determined as described before [36], and *A*_340_ was measured after 30-, 60-, and 80-minute incubation at 30°C using a spectrophotometer. Aggregates diluted with the refolding buffer B without the chaperones were used as control.

The chaperone-mediated reactivation of aggregated G6PDH and Fda was monitored in the absence of an ATP regenerating system. Therefore, to avoid significant ATP depletion and ADP accumulation, we limited the measurements to an initial stage of the reaction.

The inclusion bodies (IBs) of β-galactosidase (VP1LAC protein; *E*. *coli* β-galactosidase fused to the aggregation-prone VP1 capsid protein of the foot-and-mouth disease virus) overproduced from pJVP1LAC [37] in *E*. *coli* strains MC4100*ΔclpB*::kan or MC4100*clpB*^*+*^ (used as wild-type control; wt) and purified as described before [38] were resuspended in Z buffer (60 mM Na_2_HPO_4_, 40 mM NaH_2_PO_4_, 10 mM KCl, 1 mM MgSO_4_, 50 mM β-mercaptoethanol, pH 7.0), mixed and pipetted up and down. Subsequently, 5 mM ATP and, in the case of IBs isolated from *ΔclpB* mutant, also 1 μM ClpB_Li_ or ClpB_Ec_ were added. The β-galactosidase activity was determined after 60-minute incubation at 30°C according to Miller’s method [39]. For the calculation of units of β-galactosidase, *A*_420_ was measured using a spectrophotometer and the enzyme activity was calculated as follows: β-galactosidase (Units/ml) = (*A*_420_)/(0.0045) x (1) x (15) [40], where 0.0045, 1 and 15 indicate, the molecular extinction coefficient of *o*-nitrophenol, cuvette pathlength (cm), and the reaction time (min), respectively.

*E*. *coli* strain MC4100 (SG20250) (*ara*D139, *Δ*(*argF*-*lac*)*U*169, *rpsL*150, *rel*A1, *deo*C1, *pts*F25, *rps*R, *flb*B53010) was obtained from S. Gottesman (National Cancer Institute, Bethesda, MD), and its derivative MC4100*ΔclpB*::kan was supplied by A. Toussaint (Université Libre de Bruxelles, Brussels, Belgium). Plasmid pJVP1LAC was kindly provided by García-Fruitós (Universitat Autonòma de Barcelona, Spain).

### ClpB-aggregate interaction assay

The filtration assay was performed as reported earlier [36]. Aggregated G6PDH (21 μM) was diluted 10-fold by the addition of the refolding buffer B containing ClpB_Li_ (0.65 μM) and 5 mM nucleotide: ADP, ATP, ATPγS, or AMP-PNP. The mixtures were incubated with shaking at 30°C for 10 min and then applied to the filter devices (Millipore Ultrafree-MC Centrifugal Filter Unit with the membrane pore size 0.1 μm). After 5 min incubation at room temperature, the filter devices were centrifuged at 7,500 g for 5 min to get the flow-through fractions, then washed with the refolding buffer containing an appropriate nucleotide at 30°C for 5 min and re-centrifuged. Next, SDS-loading buffer (2x) was added to the filter devices and they were incubated at 50°C for 5 min with shaking. Then, the filter devices were centrifuged to obtain the eluate fractions, which were separated by 0.1%SDS-10%PAGE and stained with Coomassie blue dye. The stained gels were scanned and analyzed with 1Dscan EX, Scananalytics Inc. Sigma program.

### Heat-shock survival assay

The *clpB*_*Li*_ gene was cloned into a low-copy pGB2 plasmid together with the native *E*. *coli clpB* heat-shock promoter (i.e. the σ^32^- dependent promoter). The nucleotide sequence of *clpB*_*Li*_ was amplified from genomic DNA of *L*. *interrogans* by PCR using AccuTaq LA polymerase MIX (Sigma) with the following PCR primers: CATATGAAATTAATAAA CTTACATCCAAATT with the NdeI restriction site underlined, and AAGCTTTTAAA CTACAACAACTACCTTTCCCT with the HindIII restriction site underlined. The *E*. *coli* σ^*32*^ promoter was amplified from pGB2-ClpB_Ec_ [41] using the following primers: CCCGGGTTCTCGCCTGGTTAGGGC with the XmaI site underlined, and CATATG AACTCCTCCCATAACGGATC with the NdeI site underlined. First, the PCR products were cloned into pJET1.2 blunt vector (Fermentas), then digested with NdeI, HindIII, and XmaI, and ligated with the linearized pGB2/XmaI-HindIII vector to produce pGB2-ClpB_Li_. The *E*. *coli* MC4100*ΔclpB* cells were transformed with the empty pGB2, pGB2-ClpB_Ec_ [41], or pGB2-ClpB_Li_ and bacterial survival during heat-shock was determined as described earlier [41]. To detect ClpB in *E*. *coli* cultures, Western blotting was performed according to [42] using anti-ClpB_Li158-334_ serum [18], that recognized both ClpB_Li_ and ClpB_Ec_, a peroxidase-coupled goat anti-rabbit secondary antibody (Sigma), and visualized with the substrate chromogen, 3,3’-diaminobenzidine tetrachloride (DAB, Sigma) and 30% H_2_O_2_.

## Results

### Secondary structure and thermal stability of ClpB_Li_

First, we estimated the secondary structure of the recombinant ClpB_Li_ and its thermal stability by performing CD spectroscopy, which was a prerequisite for further characterization of the chaperone. As shown in Fig 1B, the CD spectrum recorded at 20°C showed local minima at 208 and 222 nm, which indicates that the recombinant ClpB_Li_ is folded into a structure that is dominated by α-helices. This result is in agreement with the secondary structure of ClpB_Ec_ obtained from spectroscopic measurements [25] and that observed in the crystal structure of ClpB from *T*. *thermophilus* [3]. Furthermore, the structure of ClpB_Li_ is thermodynamically stable at the assay temperatures used in this study, as shown by a cooperative unfolding transition (Fig 1C), with T_m_ of approx. 67°C. The thermal unfolding of ClpB_Li_ is accompanied by a loss of the α-helical structure (see dotted line in Fig 1B).

### Nucleotide-induced oligomerization of ClpB_Li_

**Fig 2:**
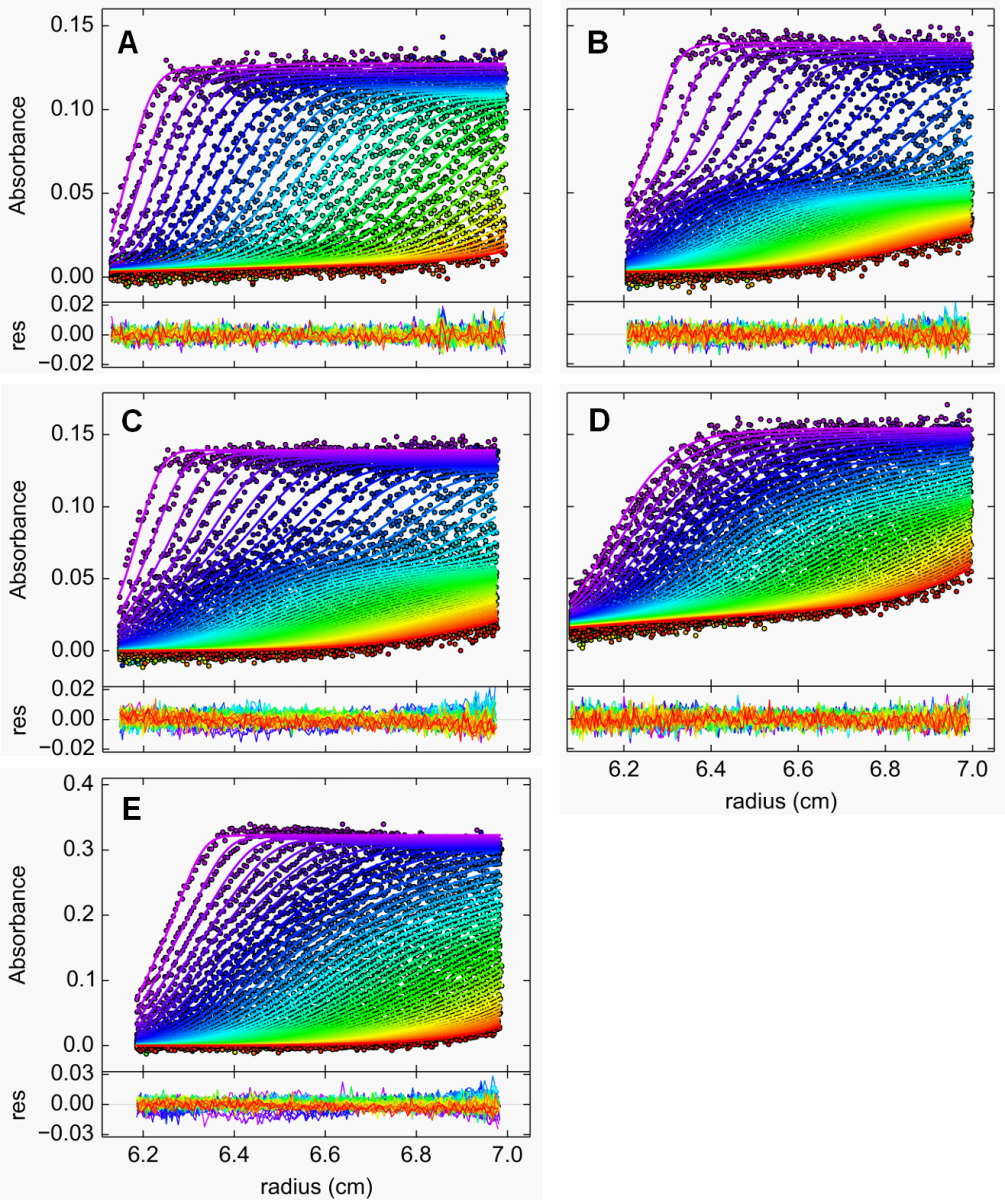
Sedimentation velocity experiments of ClpB_Li_. Radial absorption profiles at 290 nm (•) with the best fits of SEDFIT *c*(*s*) model (―) are shown for 1.2 mg/ml (A) and 3 mg/ml ClpB_Li_ (E) without nucleotides, and for 1.2 mg/ml ClpB_Li_ with 2 mM nucleotide: ATPγS (B), ADP (C) or AMP-PNP (D). Ultracentrifugation was performed at 50,000 rpm and 20°C. Radial profiles were measured at 4.5-min (A, B, C, E) or 5-min (D) intervals in 50 mM Tris-HCl buffer pH 7.5 containing 20 mM MgCl_2_, 2 mM β-mercaptoethanol, 1 mM EDTA, 5% glycerol and 200 mM (A-D) or 30 mM KCl (E). For (A) every second profile is shown for clarity. Bottom panels present the fitting residuals. The time evolution of radial distributions was plotted as colored curves in the order of purple-blue-green-yellow-red.

**Fig 3:**
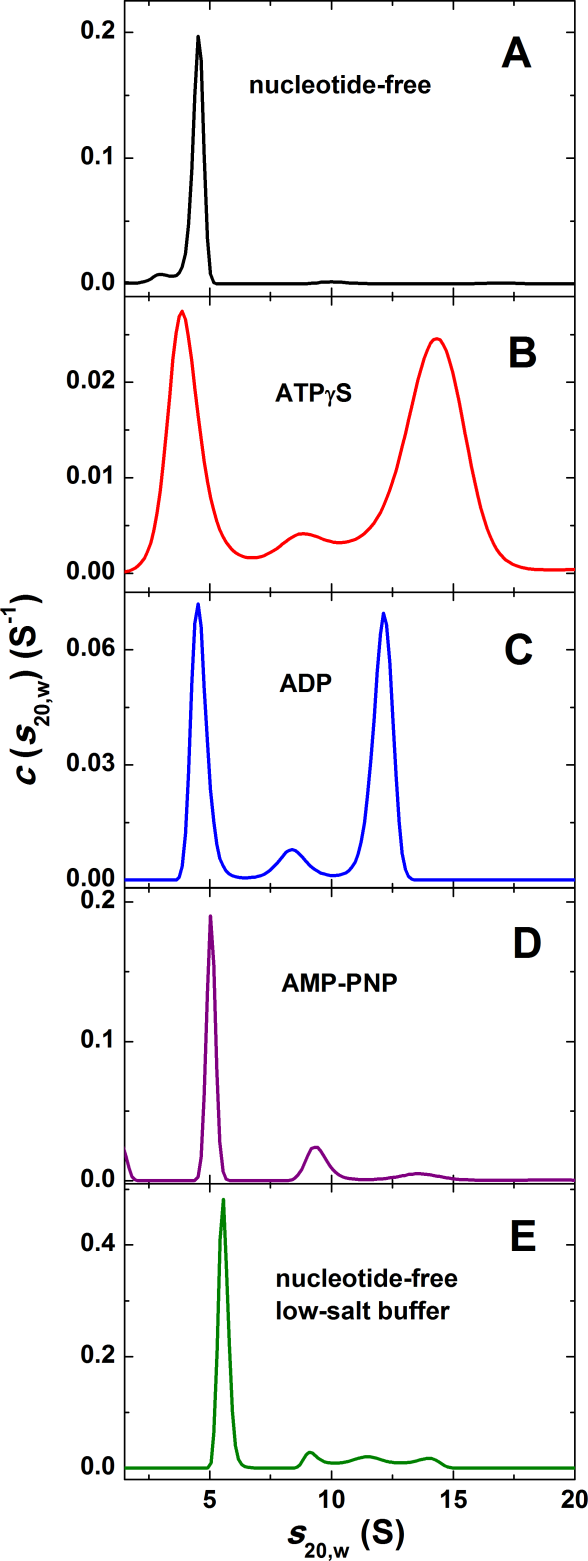
Nucleotide-induced oligomerization of ClpB_Li_. Shown are the sedimentation coefficient distributions *c*(*s*_20,w_) for 1.2 mg/ml ClpB_Li_ in the absence of nucleotides (A), in the presence of the indicated nucleotide at 2 mM concentration (B-D), and in the low-salt buffer without nucleotides for 3 mg/ml ClpB_Li_ (E). Sedimentation velocity data presented in Fig 2 were analyzed with a continuous sedimentation coefficient distribution *c*(*s*) model. The distributions were transformed to standard conditions.

It has been shown that the self-association of ClpB from *E*. *coli* (ClpB_Ec_) into hexameric ring-shaped structures is tightly regulated by protein concentration and enhanced by the presence of nucleotides [9,43]. It has been also shown that hexamerization of ClpB_Ec_ is necessary for its ATPase activity and the biological function [25]. Therefore, we decided to study the self-assembly of the recombinant ClpB_Li_ and answer the question whether it forms nucleotide-induced oligomers. For this purpose, we carried out sedimentation velocity experiments (Figs 2 and 3).

As shown in Fig 3A, ClpB_Li_ in the absence of nucleotides sedimented as a single predominant species with the standard sedimentation coefficient *s*_20,w_ = 4.5 S, which agreed with the previously determined sedimentation coefficient of the monomeric ClpB_Ec_ [25]. The addition of a weakly hydrolyzed ATP analogue, ATPγS (Fig 3B), but not AMP-PNP (Fig 3D) induced efficient self-association of ClpB_Li_, manifested by the presence of a major peak at 14.1 S in the sedimentation coefficient distribution. The maxima of the distribution are broad, indicating that several types of oligomers are in rapid equilibrium in the presence of ATPγS. In such cases, the peaks appear at intermediate positions, which do not correspond to the *s*-values of the sedimenting species [44]. The value of 14.1 S is lower but close to the sedimentation coefficient of the hexameric ClpB_Ec_ [9]. Thus, we conclude, that in the presence of ATPγS, the hexamer of ClpB_Li_ is most likely the largest oligomeric species. As shown in Fig 3C, in the presence of ADP, two major components are observed with sedimentation coefficients ~4.6 S and ~12.1 S. However, the position of the fastest sedimenting peak is about 2 S lower than for the distribution in the presence of ATPγS. This result suggests that ADP does not support full association of ClpB_Li_. Since a high salt concentration is known to promote dissociation of subunits in oligomeric proteins, we tested whether a low salt concentration may stabilize ClpB hexamers without nucleotides present. Fig 3E shows the sedimentation velocity result obtained for ClpB_Li_ (at a concentration of 3 mg/ml) in a buffer with 30 mM KCl (vs. 200 mM KCl in panels A-D). We found that low salt concentration and an increased ClpB_Li_ concentration did not result in efficient self-association of the chaperone, because ClpB_Li_ sedimented primarily at 5.6 S.

Taken together, our observations (Fig 3 and Table 1) indicate that the ATP analogue, ATPγS, but not ADP, induces an efficient self-association of ClpB_Li_ into hexamers. ADP does induce oligomerization of ClpB_Li_, but less efficiently than ATPγS.

**Table 1:** Summary of sedimentation coefficients for ClpB_Li_.

ATP analogue [2 mM]	ClpB_Li_ [mg/ml]	*s*_20,w_ [S] (population %)			
		peak 1	peak 2	peak 3	peak 4
–	1.2	4.5 ± 0.3 (86%)	10.2 ± 0.7 (2%)	–	16.9 ± 1.0 (<1%)
–	3^**a**^	5.6 ± 0.2 (74%)	9.5 ± 0.4 (9%)	11.6 ± 0.6 (10%)	13.7 ± 0.4 (7%)
*γ*-S	1.2	4.1 ± 0.8 (33%)	8.8 ± 1.0 (8%)	–	14.1 ± 1.4 (55%)
ADP	1.2	4.6 ± 0.4 (41%)	8.4 ± 0.7 (9%)	12.1 ± 0.5 (50%)	–
AMP-PNP	1.2	5.1 ± 0.2 (61%)	9.5 ± 0.5 (19%)	–	13.7 ± 1.0 (8%)

The *s*_20,w_-values (mean ± S.E.) were determined from the data shown in Figs 2 and 3. Signal-weighted average sedimentation coefficients obtained by integration of the *c*(*s*) distribution peaks were corrected to standard conditions to provide the standard sedimentation coefficients *s*_20,w_ (corresponding to the density and viscosity of water at 20°C).

^a^, The values determined for the low concentration of KCl (30 mM instead of 200 mM) in 50 mM Tris-HCl buffer pH 7.5 containing 20 mM MgCl_2_, 2 mM β-mercaptoethanol, 1 mM EDTA, 5% glycerol.

**Fig 4:**
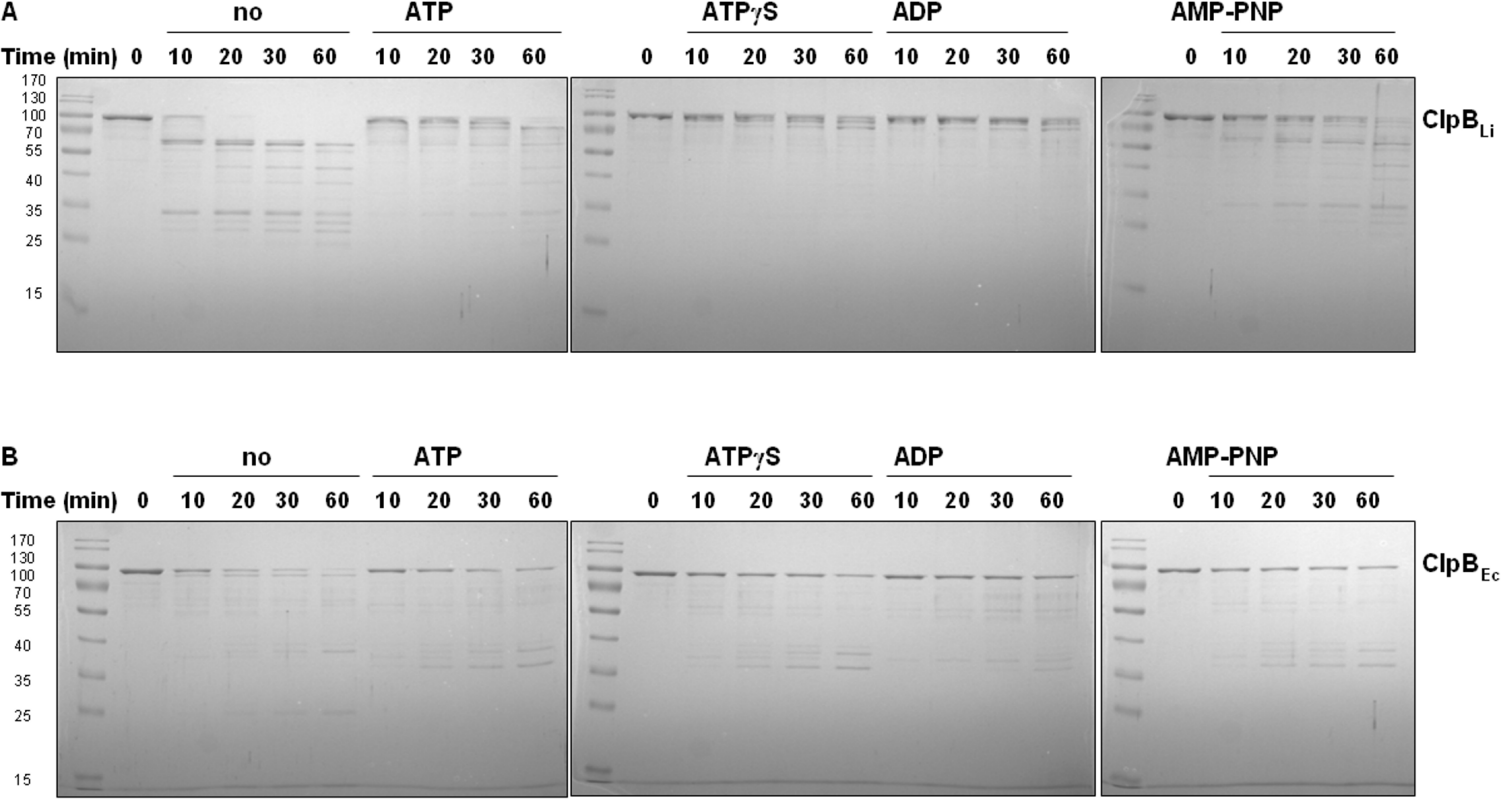
Proteolytic sensitivity of ClpB_Li_ and ClpB_Ec_ in the absence and presence of nucleotides. ClpB (1 μM) was incubated at 37°C for the indicated periods with 5 ng of trypsin in the absence or presence of 5 mM nucleotides. The degradation products were resolved by 0.1%SDS-12.5%PAGE and visualized by Coomassie-blue staining. Representative results from three experiments are shown. The positions of standard molecular mass markers (M) (in kDa), PageRuler prestained Protein Ladder (ThermoScientific), are shown on the left.

We also investigated the effect of nucleotides on the structure of ClpB_Li_ by monitoring changes in its proteolytic degradation (Fig 4A). We compared the proteolytic pattern obtained for ClpB_Li_ with that found for ClpB_Ec_ (Fig 4B). The ClpB proteins were digested with trypsin in the absence of nucleotides and in the presence of ATP, ATPγS, ADP and AMP-PNP. Subsequently, the degradation products were separated by SDS-polyacrylamide gel electrophoresis and stained with Coomassie blue dye (Fig 4A and 4B). We found that in the absence of nucleotides, ClpB_Li_ was digested into several fragments in the ~25- to ~60-kDa range with a complete loss of the intact ~100-kDa ClpB_Li_ after 20 min of incubation with trypsin. In the presence of nucleotides, ClpB_Li_ showed varying degrees of protection against trypsin digestion, which indicates that ClpB_Li_ undergoes structural changes upon binding of all tested nucleotides. Interestingly, ClpB_Li_ in the presence of either ATPγS or ADP was more resistant to proteolysis than ClpB_Ec_, (compare Fig 4A and 4B), as shown by a lack of prominent digestion fragments in ClpB_Li_ with ATPγS and ADP and their presence in ClpB_Ec_.

A protective effect of nucleotides on ClpB_Li_ was weaker in the presence of ATP or AMP-PNP than with ATPγS or ADP, as shown by low-molecular weight fragments appearing after 10–20 min of incubation with trypsin. A partial loss of protection against trypsin for ClpB_Li_ with ATP, as compared to the ATPγS-bound state, can be due to a fraction of nucleotide-free ClpB_Li_ populated during the ATP turnover. ADP does induce oligomerization of ClpB_Li_, but not as efficiently as ATPγS (Fig 3B and 3C). Apparently, an incomplete oligomerization induced by ADP provides ClpB_Li_ with a significant protection against trypsin, comparable to that of ATPγS. As for AMP-PNP, a lower extent of the ClpB_Li_ protection can be explained by a low population of the ClpB_Li_ oligomers induced by that ATP analogue (Fig 3D). Overall, the extent of the ClpB_Li_ protection against trypsin in the presence of different nucleotides (Fig 4) correlates with the capability of those nucleotides to stabilize the ClpB_Li_ oligomers (Fig 3).

### ATPase activity of ClpB_Li_

**Fig 5:**
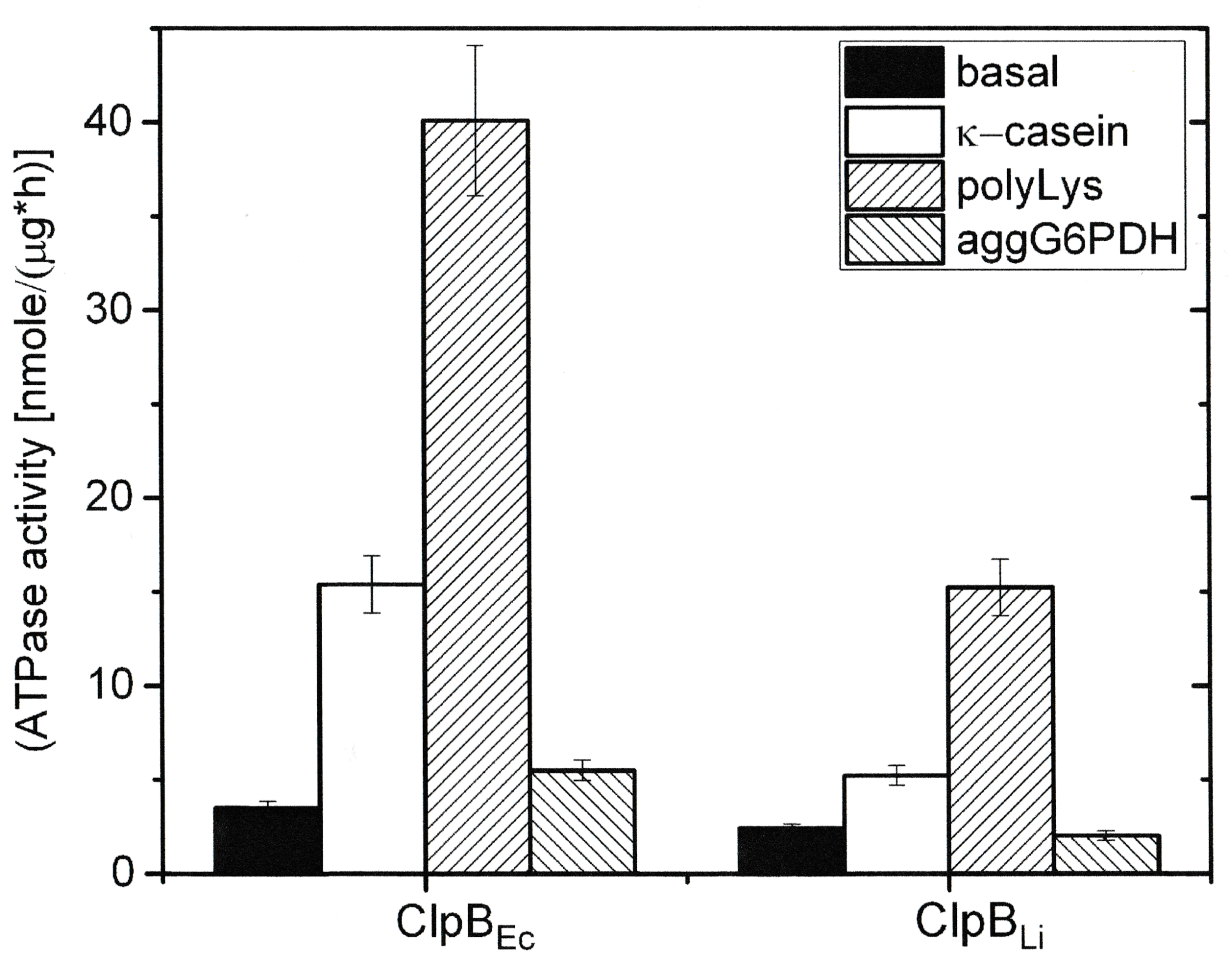
ATPase activity of ClpB_Li_ and ClpB_Ec_. The rate of ATP hydrolysis was determined at 37°C in the absence of other proteins (basal activity), in the presence of κ-casein (0.1 mg/ml), poly-lysine (0.04 mg/ml) (polyLys), or aggregated G6PDH (2.1 μM) (aggG6PDH). The average values from three independent experiments are shown with the standard deviations.

We determined the ATPase activity of ClpB_Li_ under the same conditions as the previously tested ClpB_Ec_ [25,36] using a malachite green phosphate-detection assay. As shown in Fig 5, ClpB_Li_ exhibits a slightly lower basal ATPase activity than ClpB_Ec_. The presence of unstructured polypeptides: κ-casein or poly-lysine, which are known to enhance the ATPase activity of ClpB_Ec_ up to 5- and 25-fold, respectively [45], caused an increase in the ATPase activity of ClpB_Li._ Thus, the ATPase of ClpB_Li_ resembles in this respect the ATPase of ClpB_Ec_. Aggregated glucose-6-phosphate dehydrogenase (G6PDH) did not significantly affect the ATPases of ClpB_Li_ and ClpB_Ec_.

### Chaperone activity of ClpB_Li_

It is known that ClpB_Ec_ efficiently reactivates aggregated proteins in cooperation with DnaK/DnaJ/GrpE [1,25]. Therefore, we tested the ability of ClpB_Li_ to reactivate aggregated protein substrates in the presence of the *E*. *coli* DnaK chaperone system. In the reactivation assays, we used chemically denatured G6PDH that was previously tested *in vitro* as a ClpB substrate [4,36] and two new model substrates: thermally aggregated FBP aldolase (Fda) and inclusion bodies (IBs) of VP1-β-galactosidase (VP1LAC). Using the pull-down strategy coupled with the mass spectrometry (MS) analysis, we identified Fda as a putative substrate for ClpB_Li_ (unpublished data). As we reported earlier, ClpB_Ec_ significantly increased the reactivation yield of β-galactosidase aggregated in the form of IBs [38,46].

**Fig 6:**
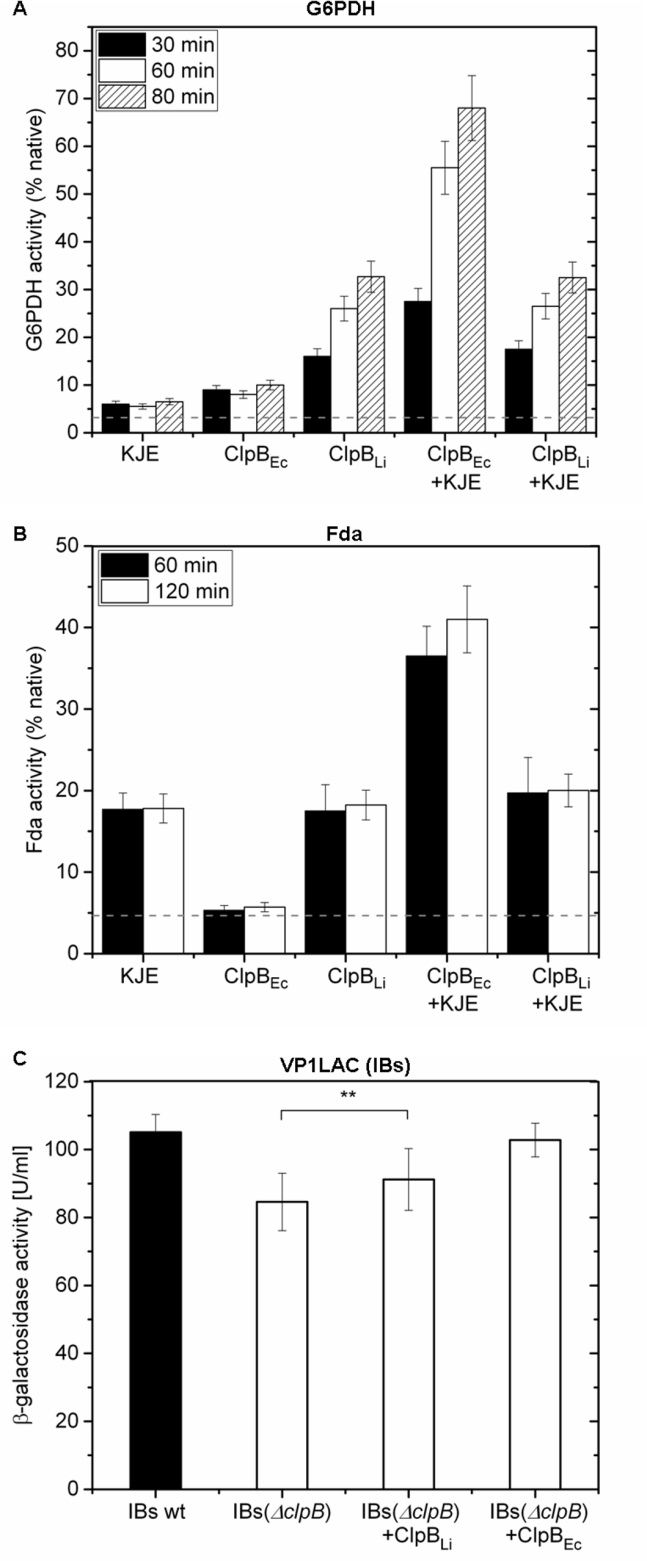
Reactivation of the aggregated substrates in the presence of ClpB_Li_ and ClpB_Ec_. The reactivation of aggregated enzymes, G6PDH (A) and Fda (B) in the presence of DnaK/DnaJ/GrpE (KJE) from *E*. *coli* without ClpB and with ClpB_Ec_ or ClpB_Li_. The native activity of G6PDH or Fda determined before the chemical denaturation or the heat treatment at 55°C, respectively, corresponds to 100%; the fraction of the enzyme activity remaining after the denaturation and also corresponding to the reactivation extent in the absence of chaperones (control) is marked by the broken line. (C) The effect of ClpB_Li_ and ClpB_Ec_ on the reactivation of β-galactosidase sequestered into IBs (VP1LAC) isolated from *E*. *coliΔclpB* mutant cells. A statistically significant difference in the β-galactosidase activity regain in the absence and presence of ClpB_Li_ assessed by the paired t-test (using GraphPad Prism software) is indicated as **, p<0.01. The results are presented as the average of three (A, C) or four (B) independent experiments with the standard deviations indicated.

The reactivation yield for the aggregated G6PDH (Fig 6A) in the presence of ClpB_Li_ was significantly higher than for ClpB_Ec_ or the *E*. *coli* DnaK/DnaJ/GrpE system. In the case of reactivation of the aggregated Fda (Fig 6B), ClpB_Li_ was again more effective than ClpB_Ec_ and equally potent as DnaK/DnaJ/GrpE. These results demonstrate an intrinsic disaggregase activity of ClpB_Li_, which, for the selected substrates, exceeds that of ClpB_Ec_. However, the aggregate reactivation yields in Fig 6A and 6B obtained with ClpB_Li_ in the presence of the *E*. *coli* DnaK/DnaJ/GrpE were similar to those obtained with ClpB_Li_ alone. This result suggests that ClpB_Li_ does not interact with the *E*. *coli* DnaK chaperone system during aggregate reactivation *in vitro*. The reactivation of β-galactosidase from IBs produced in *E*. *coli* (Fig 6C) occurred more efficiently with ClpB_Li_ than in the absence of ClpB, but did not reach the efficiency of ClpB_Ec_. This observation, again, suggests that ClpB_Li_ is capable of reactivating protein aggregates, but does not cooperate with the *E*. *coli* co-chaperones. Altogether, these properties of ClpB_Li_ are similar to the previously investigated ClpB from a zoonotic bacterium *Ehrlichia chaffeensis* [36].

### Interaction of ClpB_Li_ with aggregated protein substrates

**Fig 7:**
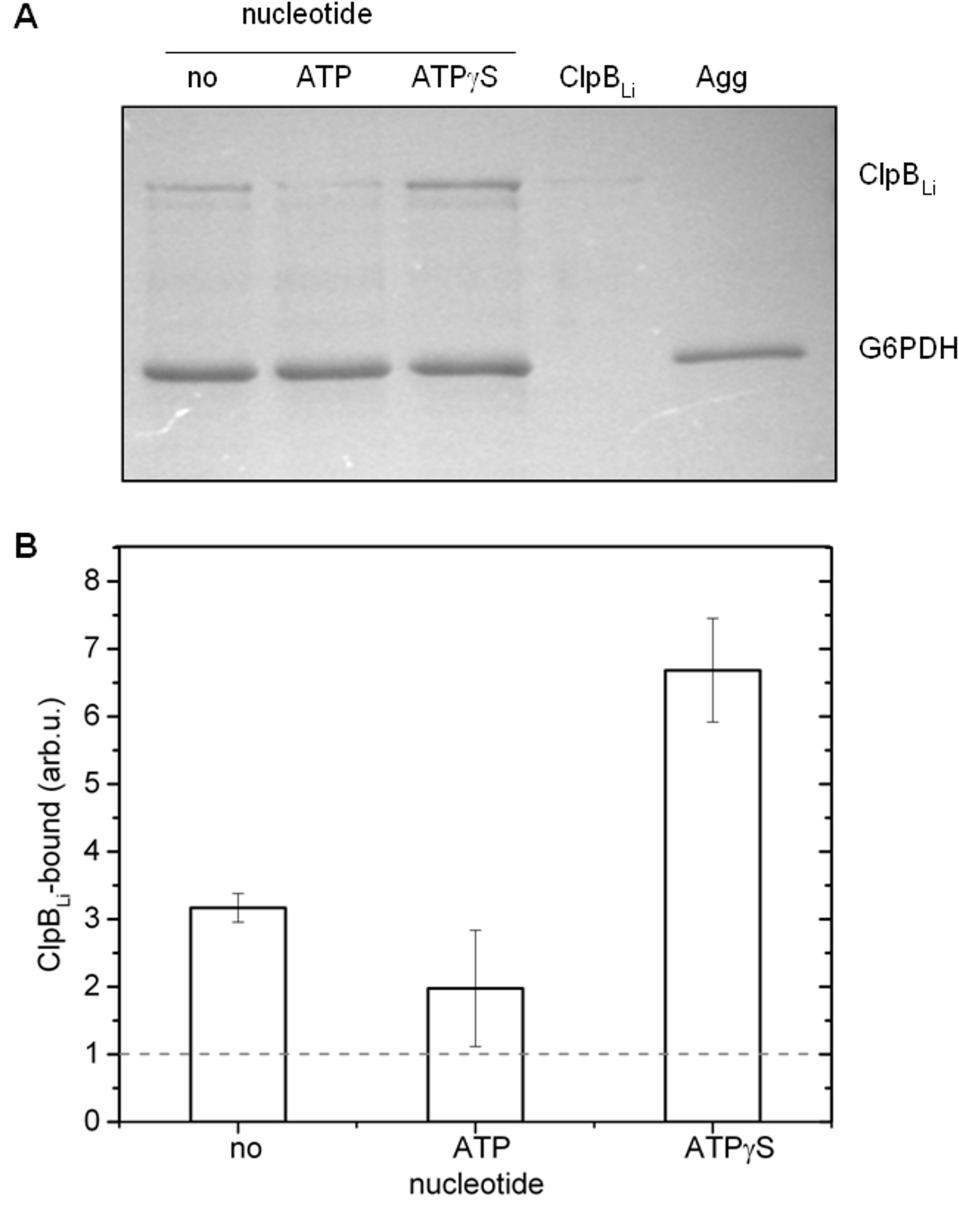
Interaction of ClpB_Li_ with the aggregated G6PDH. (A) ClpB_Li_ was incubated with aggregates of G6PDH (Agg) in the presence of 5 mM ATP or ATPγS and without nucleotides. The solutions were passed through a 0.1-μm filter. Subsequently, the fractions retained on the filters were solubilized with an SDS buffer and analyzed by the Coomassie blue-stained 0.1%SDS-10%PAGE gel. A representative result from three independent experiments is shown. (B) Bands corresponding to ClpB_Li_ were analyzed with Sigma Gel software. Results are presented as the average of three independent experiments with standard deviations indicated. The amount of ClpB_Li_ detected in the absence of the aggregates is indicated with the broken line.

Previous studies demonstrated that nucleotides regulate interactions of bacterial ClpBs with the aggregated substrates. It has been shown that only ATPγS promotes significant binding of ClpB_Ec_ to the aggregates [13,36], while ClpB from *E*. *chaffeensis* interacts more efficiently with aggregates in the presence of the hydrolysable ATP [36]. Other nucleotides, such as ADP and AMP-PNP did not induce significant binding of ClpB_Ec_ to protein aggregates. We tested whether binding of ClpB_Li_ to the aggregates is more efficiently stimulated by ATPγS (which induces a “frozen” ATP-like ClpB state) or by the hydrolysable ATP. We incubated ClpB_Li_ with the aggregated G6PDH in the absence and presence of the tested nucleotides and then determined the amount of ClpB_Li_ bound to aggregates (Fig 7) by performing a filtration assay (see Materials and methods). Only background amounts of ClpB_Li_ were detected in the absence of the aggregates. We found that interactions of ClpB_Li_ with the aggregated G6PDH were more effective in the presence of ATPγS than in the presence of ATP, similar to ClpB_Ec_. Under the conditions of ATP turnover, ClpB_Li_ appears to lose the capability of binding stably to protein aggregates.

### Heat-shock survival of the *clpB-*null *E*. *coli* in the presence of ClpB_Li_

**Fig 8:**
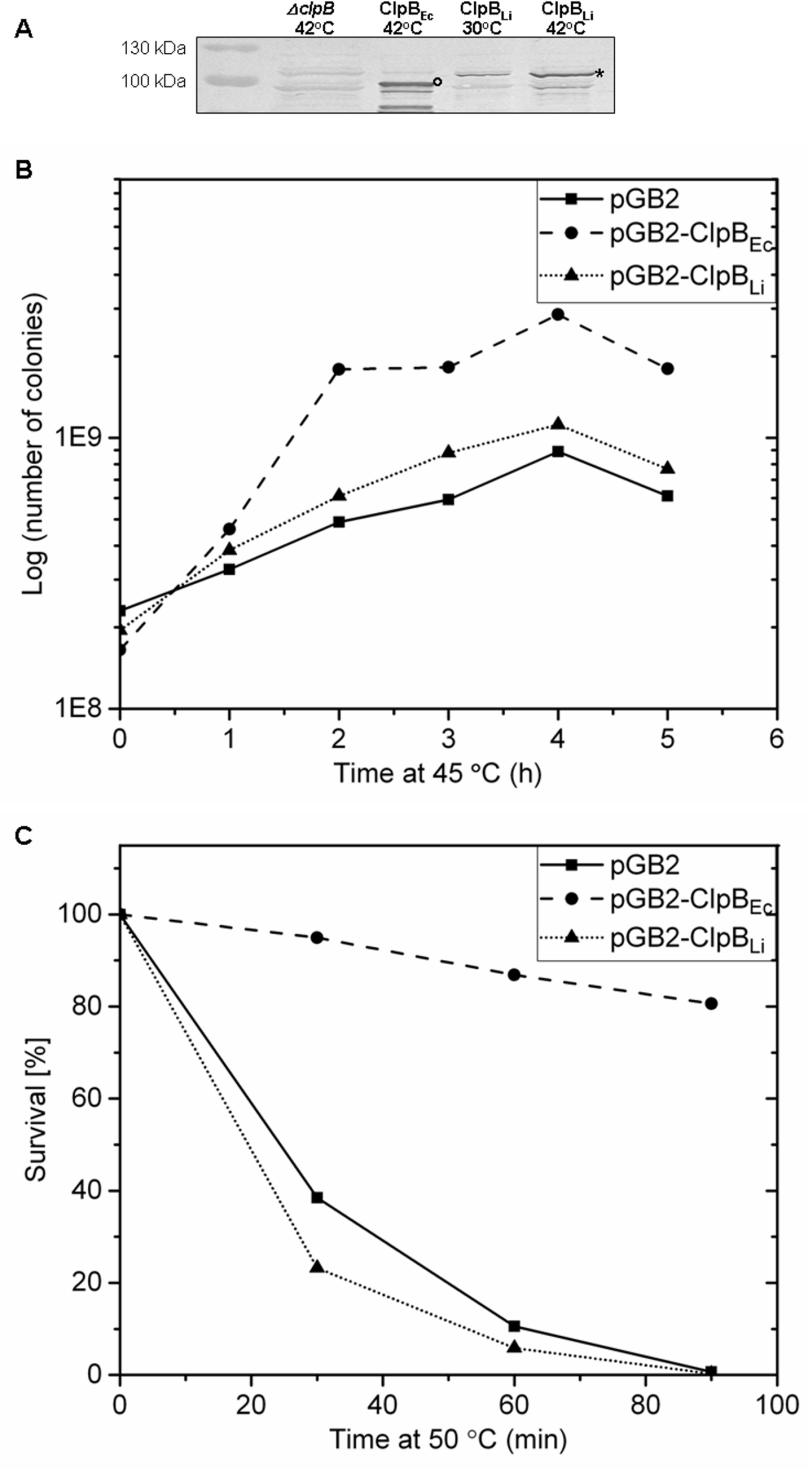
Effect of the *clpB*_Li_ gene expression on the growth and survival of *E*. *coli* *ΔclpB* mutant under heat shock. (A) Immunodetection of ClpB_Li_ with specific antibodies in *E*. *coliΔclpB* cells grown at 30°C and after 2h of heat shock at 45°C. An asterisk indicates ClpB_Li_. The position of ClpB_Ec_ (control of heat-inducible expression) was marked by a circle. (B) Growth curves of *E*. *coliΔclpB* cells carrying empty pGB2 (control 1), pGB2-ClpB_Ec_ (control 2) or pGB2-ClpB_Li_ exposed to a mild heat shock at 45°C for the indicated times. (C) Survival of the same bacterial strains as in (B) after exposure to a severe heat shock at 50°C for the indicated times. The average values from three independent experiments are shown in (B) and (C).

In *E*. *coli*, ClpB is necessary for bacterial survival under heat shock [12]. It was demonstrated that the lack of a functional ClpB decreased the growth rate of *E*. *coli* at 45°C and inhibited cell survival at 50°C. We investigated whether ClpB_Li_ can function in the *E*. *coli* cells and substitute for ClpB_Ec_. We cloned the *clpB*_*Li*_ gene into a low-copy pGB2 plasmid together with the native *E*. *coli* σ^32^-dependent *clpB* heat shock promoter. Next, the resulting plasmid pGB2-ClpB_Li_ and the control plasmids, pGB2 and pGB2-ClpB_Ec_ were introduced into the *E*. *coli ΔclpB* mutant and a heat-shock survival assay was performed (see Materials and methods). As shown in Fig 8A, the heat-inducible expression of pGB2-ClpB_Li_ produced a in the ~100-kD protein detectable by Western blotting using anti-ClpB_Li158-334_ serum [18]. We observed, however, that ClpB_Li_ was unable to functionally substitute for ClpB_Ec_ and consequently, it did not rescue *E*. *coli ΔclpB* mutant under heat shock conditions (Fig 8B and 8C). In contrast, the expression of the *clpB*_*Ec*_ gene from pGB2 complemented the effect of the *ΔclpB* mutation. With ClpB_Ec_, ~80% of the bacteria survived a severe heat shock at 50°C for 90 minutes, which is consistent with the previous results [12].

## Discussion

To date, little is known about the structure and biological role of molecular chaperones from *Leptospira* spp., including ClpB. It has been demonstrated that the *L*. *interrogans* ClpB is essential for the pathogen survival under stress conditions and also during infection of the host [18]. Moreover, a recently reported immunoreactivity of ClpB_Li_ with the sera collected from *Leptospira*-infected animals [24] and the fact that *clpB*_*L*i_ is up-regulated in leptospiral cells [18] suggest that the ClpB activity may be important for pathogenicity of *Leptospira*. In this work, we described the biochemical and structural properties of ClpB from *L*. *interrogans*.

As demonstrated by the sedimentation velocity experiments (Figs 2 and 3), ClpB_Li_ forms hexamers in the presence of the ATP analogue, ATPγS (Fig 3B), while it exists as a monomeric protein in the absence of nucleotides (Fig 3A). In contrast to ATPγS, ADP induces partial self-association of ClpB_Li_ (Fig 3C). Insofar as ATPγS-induced hexamerization is rather typical for bacterial ClpB homologues, the effect of ADP on their oligomerization *in vitro* appears species-dependent. Specifically, ADP stabilized the hexameric forms of the yeast ClpB orthologue, Hsp104 [47,48] or ClpB from the halophilic lactic acid bacterium *Tetragenococcus halophilus* [49], whereas ClpB_Ec_ [43] and the *T*. *thermophilus* ClpB [50] did not fully assemble into hexamers in the presence of ADP, similar to our results for ClpB_Li_. Unexpectedly, another ATP analogue, AMP-PNP, did not induce an effective assembly of oligomeric ClpB_Li_ (Fig 3D). This result indicates that AMP-PNP binding inhibits self-association of ClpB_Li_ or that the affinity of AMP-PNP towards ClpB_Li_ is low. A similar effect, where AMP-PNP did not induce oligomerization was observed for ClpB from *Tetragenococcus halophilus* [49]. We also tested the effect of a low salt concentration on the ClpB_Li_ oligomerization in the absence of nucleotide and at an increased concentration of the chaperone (Fig 3E). In contrast to ClpB_Ec_ [51], a decreased salt concentration did not stabilize the ClpB_Li_ oligomers. The results presented in Fig 4A show a protective effect of nucleotides on ClpB_Li_ during trypsin digestion with the strongest effect for ATPγS and ADP and a weaker one with AMP-PNP, which correlates with the extent of oligomerization induced by these nucleotides (see Fig 3).

Like other previously characterized ClpB proteins, ClpB from *L*. *interrogans* catalyzes the hydrolysis of ATP (Fig 5), stably binds to aggregated substrates in the presence of an ATP analogue (Fig 7), and shows a disaggregase activity towards aggregated proteins: G6PDH, Fda and β-galactosidase trapped in IBs (Fig 6). It is worth noting that the reactivation yield of Fda (see Fig 6B) in the presence of ClpB_Li_ alone was similar to that obtained with the *E*. *coli* DnaK chaperone system, but ClpB_Li_ was significantly more efficient in mediating the aggregate reactivation than ClpB_Ec_. It has been previously shown that the DnaK chaperone system can disaggregate some substrates, specifically small-size and low-complexity aggregates [28,52,53]. Thus, the intrinsic disaggregase activity of ClpB_Li_ in Fig 6A and 6B manifesting in the absence of the co-chaperones and exceeding that of ClpB_Ec_ suggests that the range of potential aggregated substrates of ClpB_Li_ may be broader than for ClpB_Ec_ and may overlap with that of DnaK.

Furthermore, the DnaK chaperone system from *E*. *coli* increased the efficiency of aggregate reactivation mediated by ClpB_Ec_, but not ClpB_Li_ (see Fig 6A and 6B). A similar effect was observed before for ClpB from a parasite *Plasmodium falciparum* [8]. The apparent lack of cooperation between ClpB_Li_ and the *E*. *coli* co-chaperones *in vitro* (Fig 6) is consistent with the result of an *in vivo* assay presented in Fig 8 (see panels B and C), which shows that ClpB_Li_ is unable to restore the viability of *E*. *coli ΔclpB* cells after heat shock. This property of ClpB_Li_ resembles ClpB from *E*. *chaffeensis*, for which, however, the *E*. *coli* DnaK system potentiated the chaperone activity during reactivation of aggregated proteins *in vitro* [36]. The apparent lack of an efficient cooperation of ClpB_Li_ with *E*. *coli* co-chaperones during protein disaggregation both *in vivo* and *in vitro* is likely due to the species-specificity of multi-chaperone networks, as reported before [5,6,8]. As has been previously shown, the middle domain of ClpB is responsible for the species-specific cooperation among the chaperones [6]. The sequence identity between ClpB_Li_ and ClpB_Ec_ within the middle domain is only ~45% (Fig 1A), which is apparently insufficient to support a cooperation between ClpB_Li_ and the *E*. *coli* DnaK system. The lack of cooperation between ClpB_Li_ and the *E*. *coli* co-chaperones also possibly explains an inability of ClpB_Li_ to rescue the survival of *E*. *coli* under heat shock (Fig 8, panels B and C). However, the results in Fig 8 could be also explained by a lower potency of ClpB_Li_ towards proteins aggregated in heat-shocked *E*. *coli* vs. those accumulating in the chaperone’s native environment of *Leptospira* during infection of the host.

## Conclusions

Our studies revealed several crucial structural and biochemical properties of the molecular chaperone ClpB from *Leptospira*, which may support its role in pathogenicity of spirochaetes. We showed that ClpB_Li_ forms hexameric assemblies that are stabilized and interact with protein aggregates in the ATP-bound state. Moreover, ClpB_Li_ exhibits a protein disaggregase activity that may contribute to the survival of *L*. *interrogans* under the host-induced proteotoxic stress. Our studies make a novel contribution to the largely uncharacterized biology of *Leptospira* and suggest that the *L*. *interrogans* and *E*. *coli* chaperones evolved differently to respond to the different nature of stress that the proteomes of either bacteria can be exposed to.
